# Supplementary material for: Chromothripsis during telomere crisis is independent of NHEJ, and consistent with a replicative origin
Source: Genome Res. 2019 May;29(5):737–49. doi: 10.1101/gr.240705.118 (PMC6499312; doi:10.1101/gr.240705.118)
Supplement: Supplemental Material [file supp_gr.240705.118_Supplemental_file_1.zip › contigs/annotated_contigs/DB77/contig.2.DB77_length_466_mean_cov_3.21888412017.docx]

**DB77_length_466_mean_cov_3.21888412017**

```
ATACATTTTTTATTAATCAGATGTGTATTTATATCTTTCAGAAAATGGATGTGAATTAAATATAGCAGCAAAAGTCAATACTTCTAGAA
 >chr12:100212451-100212663 - E=5e-116
TTCCTGTTTTTAACTGAACTTACATGTATTAGCTTAAAGCTGCAAAATATTGCAGCTATTTACATTGGATATTATTAAATTTAATTTGA

TGTTTTACTTTGTTTTTGGGAAATGGAAAG|TATT|GAATTTAGAAAAATCTCAGGCCTTCCAATCTCAGTTTTAAGGCTAGCACCCTG
                               >chr12:100198079-100198337 - E=9e-144
AACCAGAGCTTGCTTTAGAGCTTTGCCTGCATTATTTAGTAGTTTAGATATTTGTAATATCTTTTCTCAATTTCTTATTTATTCAACTT

TGGCAGCCTCTCAAAAAAGCATAAGAATTTTTTCTAACAGTCATATTAAGGAAAAACAGTCTCTCTCCTCAAAGGGGTATTGAAAATAA

GTAGGTATGCTTACTATTGTAGG
```
